# Mobile Single-Lead Electrocardiogram Technology for Atrial Fibrillation Detection in Acute Ischemic Stroke Patients

**DOI:** 10.3390/jcm11030665

**Published:** 2022-01-27

**Authors:** Marta Leńska-Mieciek, Aleksandra Kuls-Oszmaniec, Natalia Dociak, Marcin Kowalewski, Krzysztof Sarwiński, Andrzej Osiecki, Urszula Fiszer

**Affiliations:** 1 Centre of Postgraduate Medical Education, Department of Neurology and Epileptology, 00-416 Warsaw, Poland; marcin.kowalewski@cmkp.edu.pl (M.K.); ufiszer@cmkp.edu.pl (U.F.); 2 Department of Neurology and Epileptology, Professor Orlowski’s Hospital, 00-416 Warsaw, Poland; aleksandra.m.kuls@gmail.com (A.K.-O.); dociak7@tlen.pl (N.D.); 3 Department of Cardiology, Bielański Hospital, 01-809 Warsaw, Poland; krzysztof.sarwinski@gmail.com (K.S.); mcosiek@gmail.com (A.O.)

**Keywords:** mobile electrocardiography, atrial fibrillation, acute ischemic stroke

## Abstract

(1) Background: AliveCor KardiaMobile (KM) is a portable electrocardiography recorder for detection of atrial fibrillation (AF). The aim of the study was to define the group of acute ischemic stroke (AIS) patients who can use the KM device and assess the diagnostic test accuracy. (2) Methods: the AIS patients were recruited to the study. Thirty-second single-lead electrocardiogram (ECG) usages were recorded on demand for three days using KM portable device. Each KM ECG record was verified by a cardiologist. The feasibility was evaluated using operationalization criteria. (3) Results: the recruitment rate among AIS patients was 26.3%. The withdrawal rate before the start of the intervention was 26%. The withdrawal rate after the start of the intervention was 6%. KM device detected AF in 2.8% of AIS patients and in 2.2% of ECG records. Cardiologist confirmed the AF in 0.3% AIS patients. Sensitivity and specificity of KM for AF was 100% and 98.3%, respectively. (4) Conclusions: the results of this study suggest that it is feasible to use KM device to detect AF in the selected AIS patients (younger and in better neurological condition). KM detected AF in the selected AIS patients with high specificity and sensitivity.

## 1. Introduction

Thirty percent of ischemic strokes are of an unknown cause (cryptogenic). Several mechanisms are implicated with cryptogenic stroke. Nearly 65% of patients have cortical infarcts on brain imaging, a characteristic typically suggestive of embolism [1,2]. Atrial fibrillation (AF) is not only the most common cardiac arrhythmia in adults, but also the most common cause of cardioembolism resulting in ischemic stroke. Identifying individuals with AF in high-risk groups of patients, such as poststroke patients, could enable those patients to be treated properly [3,4].

AF diagnosis is difficult, especially in patients with paroxysmal AF and in asymptomatic (silent) AF. AF symptoms (history of palpitations, dyspnea, fatigue, chest tightness/pain, syncope/presyncope, and dizziness) could be absent during most episodes [4,5,6,7]. Up to 95% of patients with AF detected after stroke and transient ischemic attack (TIA) are asymptomatic [8,9,10].

Short-term electrocardiogram (ECG) recordings for at least the first 24 h after thromboembolic event, followed by continuous ECG monitoring for at least 72 h, whenever possible, are recommended in the search for AF in patients with cryptogenic stroke [4]. The ECG on admission detected new AF in 3.5–7.7% of patients, and moreover, during the hospital stay new AF was documented in another 5.1% using different routine diagnostic methods (4.4% by 6-day Holter monitoring) [11,12].

Patients in which standard diagnostic methods did not detect AF episodes might be screened using several technologies: automated blood pressure monitors, single-lead ECG devices, photopletysmography (PPG) devices, and other sensors (seismocardiography, accelerometers, and gyroscopes, etc.) used in applications for smartphones, wrist bands, and watches [13,14]. Intermittent detection of AF is possible through PPG or ECG recordings. Smartwatches and other “wearables” can passively measure pulse. To establish a definitive diagnosis of AF, a single-lead ECG tracing ≥ 30 s or 12-lead ECG showing episodes of AF lasting at least 30 s and analyzed by a physician with expertise in ECG rhythm interpretation is necessary [15].

AliveCor KardiaMobile (KM) ECG provides portable ECG recording for intermittent detection of AF, and it works with compatible mobile devices such as smartphones and tablets. It records on demand, stores, and transfers a single-channel, 30-s ECG. The KM device communicates with KM app, which can be downloaded on smartphones running Android OS or iOS. An automated algorithm on the KM app checks the ECG for RR wave irregularity. KM devices are Food and Drug Administration (FDA)-cleared to discriminate AF from sinus rhythm, and they demonstrated high sensitivity and specificity in screening studies [16,17,18]. Results from a 30-s single-lead ECG were sufficient for 42.7% of healthcare practitioners to recommend oral anticoagulation for patients with a high risk of stroke [19].

After the occurrence of ischemic stroke, the need to search for AF becomes obvious to start optimal secondary prevention. AF episodes may be missed using the standard evaluation, and therefore there is a need for improving poststroke monitoring of unknown AF detection. New technologies, such as mobile electrographic monitoring, could facilitate this in selected acute ischemic stroke (AIS) patients [20]. KM device is easy to use, noninvasive, and does not require trained healthcare staff. Its feasibility and diagnostic accuracy in AIS patients were understudied.

According to Koh et al., a 30-day smartphone ECG recording among patients with a cryptogenic stroke or TIA (index event) within the previous 12 months significantly improved the detection of AF when compared with that of the standard repeat 24-h Holter monitoring [21]. In the mentioned study, the mean duration from the index event to randomization was 87.1 days, thus patients were not in the acute phase of the disease. The data from the literature demonstrated increased detection of AF in stroke patients with earlier monitoring [22,23,24,25]. We found no literature on smartphone ECG recording to detect AF in AIS patients.

We aimed to determine the utility and feasibility of a mobile, intermittent single-channel KM ECG in hospitalized AIS patients. The aims of the study were (1) to define the group of AIS patients who can use the device and (2) to assess the diagnostic testing accuracy.

## 2. Materials and Methods

### 2.1. Ethics and Recruitment

Approval for the study by the local Ethics Committee of Centre of Postgraduate Medical Education was obtained before subject enrolment (Prot. 23/PB/2017). The study was conducted in the Department of Neurology and Epileptology, Professor Orlowski’s Hospital, Warsaw, Poland. The research team discussed the study with the patients, allowed them to read the informed consent, and answered all questions. All studied patients signed the offline consent form. Ischemic stroke was diagnosed based on the neurological syndrome and results of head computed tomography examination. The inclusion criteria for the study group were as follows: (1) AIS patient (2) at least 18 years of age, who was able (3) to use the AliveCor KM ECG monitor (i.e., they were able to record at least one 30-s ECG) and (4) to provide informed consent. The exclusion criteria were: (1) AF on the initial 12-channel ECG; (2) a history of paroxysmal or persistent AF; (3) AF on 24-h Holter ECG registered during the present hospitalization period; and (4) mechanical thrombectomy treatment for AIS. Patients with AF diagnosis (AF history or AF registered on stroke unit using standard methods) were excluded to adjust the study design to the clinical practice (searching for AF only in the group of AIS patients without AF diagnosis). Patients treated with mechanical thrombectomy were excluded, as they were transferred to the other hospital for the thrombectomy procedure.

The following demographic and clinical data were obtained via a questionnaire: age, sex, length of stay in the neurological department, history of palpitation, dyspnea, fatigue, chest tightness/pain, syncope/presyncope, and dizziness.

Stroke severity and neurological deficit measures were assessed using Scandinavian Stroke Scale (SSS), which is based on various clinical observations. The assessment was done on the 1st and 7th days after admission to the stroke unit and on the last day of the hospital stay. SSS includes nine items representing consciousness, eye movements, arm, hand, and leg motor function, orientation, speech, facial palsy, and gait. Each item has 2–5 response categories, with item scores ranging from 2 to 12. The scale total score range is between 0 and 58. Higher scores indicate better neurological function [26,27].

### 2.2. Feasibility

The feasibility was evaluated using following operationalization criteria:

- source of recruitment: at stroke unit;
- recruitment rates: from the AIS patients treated at the stroke unit, the number of eligible AIS patients was determined using inclusion and exclusion criteria (≥20%);
- withdrawal rates (before intervention): AIS patients who gave preconsent to participate but withdrew it before the start of intervention were accounted for. AIS patients for whom the device was not available were counted (≤20%);
- withdrawal rates (during the course of intervention): AIS patients who received a tablet and the KM pad for three days but for whom there was no ECG registration on the device were accounted for (≤10%);
- technical issues: problems that disrupted or impeded proper operation of the device [28,29].

### 2.3. KM ECG Monitoring

**Figure 1:**
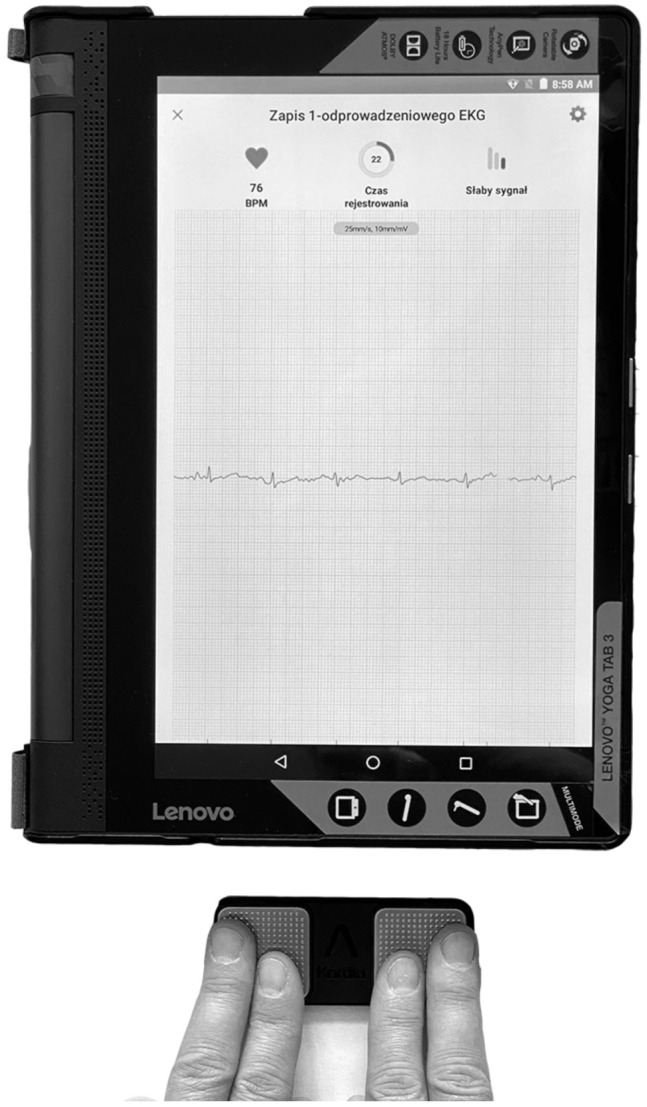
KardiaMobile (KM) portable device (AliveCor Inc, San Francisco, CA, USA) and compatible portable tablet computer (Lenovo YT3-X50L, HK Limited, China) with AliveCor application.

Thirty-second single-lead ECG was recorded using KM portable device (AliveCor Inc, San Francisco, CA, USA) and compatible portable tablet computer (LenovoYT3-850L and Lenovo YT3-X50L, HK Limited, China) with AliveCor application (Figure 1). The KM device is FDA- and Conformité Européenne (CE)-approved, registers ECG on-demand, and works with iOS and Android devices. The KM pocket-sized pad consists of two metal electrodes. When they are touched by the right and left hands of the user, a bipolar lead I is created. The cardiac electric signal is converted into an ultrasound FM sound signal (18–24 kHz). The iOS or Android device application demodulates the sound signal to a digital ECG tracing (300 samples/s, 16-bit resolution). The ECG tracing can be viewed in real-time, and it is also stored [30]. Patients randomized to the study were provided with a tablet and the KM pad for three days. Patients were trained by the Neurology Department staff members on how to use the KM card and how to record ECG on tablet. The device was used repeatedly for intermittent screening. Patients were instructed to record ECG if any symptoms of AF appeared (palpitation, dyspnea, fatigue, chest tightness/pain, syncope/presyncope, and dizziness) and several times during the day (if possible, every 2–3 h) [31]. Further tuition following the initial guidance were offered to patients, who had problems with operation of the KM device. The lead-I ECG was interpreted by an algorithm and verified by a cardiologist. The application classifies an ECG as a sinus rhythm, possible AF, or unclassified. If the registration time was shorter than 30 s, the analysis was not performed. In some cases (poor quality recordings; too much interference in the recording), the algorithm was not able to analyze ECG, even if the time of registration exceeded 30 s (analysis was impossible).

The following data from the patients’ use of the KM device were analyzed: number of times device was used, number of ECGs interpreted by algorithm as AF or unclassified, and the number of ECGs for which analysis was impossible or time of registration was too short.

### 2.4. ECG Adjudication Analysis

The KM ECG records were printed out in 1:1 ratio and assessed in paper-based form. Each of the lead-I ECG was verified by a cardiologist, and the following data were analyzed: the number of ECGs interpreted as AF, extrasystoles, bigeminy, or bundle branch block.

### 2.5. Twenty-Four-Hour Holter ECG

Standard 24-h Holter ECG was performed using BTL-08 Holter H100 3-channel Holter recorder (BTL Industries Ltd., Hertfordshire, UK). The following data were analyzed: the number of the supraventricular ectopic beat (SVEB), ventricular ectopic beat (VEB), and supraventricular tachycardia (SVT).

### 2.6. Statistical Analysis

Demographic and clinical data are reported as mean ± SD and/or median. Pearson’s correlation analyses were used to examine the associations between the number of KM ECG uses and patient’s age. Pearson’s chi-square test was used to determine whether there is a significant relationship between the AF clinical signs (history of palpitation, dyspnea, fatigue, chest tightness/pain, syncope/presyncope, and dizziness) and AF reporting during the KM device use. Comparisons are presented by showing the data in the respective groups (AliveCor algorithm/cardiologist interpretation) together with the *p* value of Pearson’s chi-square test. Mann–Whitney U test was used to correlate Holter ECG data (number of SVEB/VEB/SVT) and AF reporting during KM device use. Kendall’s tau b test was used to determine the correlation between the number of KM ECG uses and AF registration. Diagnostic accuracy measures (sensitivity, specificity) of KM for AF with corresponding 95% CI were calculated using 2 × 2 contingency tables. All statistical analyses were performed using PAWS Statistic 18 (SPSS). A *p* value < 0.05 was considered significant.

## 3. Results

### 3.1. Participants

**Figure 2:**
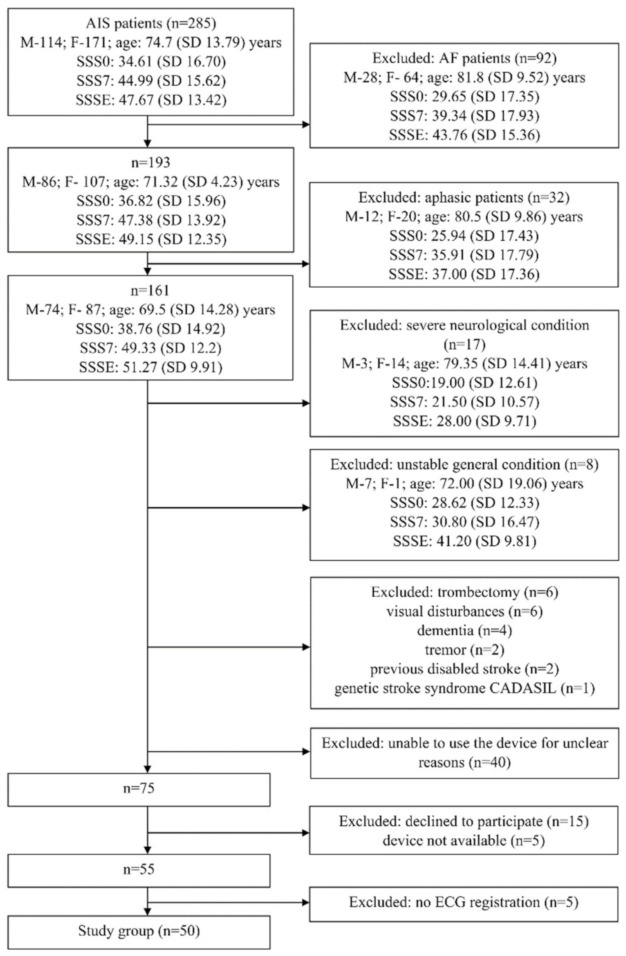
Flow diagram of study. AF—atrial fibrillation, AIS—acute ischemic stroke, ECG—electrocardiogram, SSS0—Scandinavian Stroke Scale (1st day after admission to the stroke unit), SSS7- Scandinavian Stroke Scale (7th day after admission to the stroke unit), SSSE—Scandinavian Stroke Scale (on the last day of the hospital stay).

AIS patients treated at the stroke unit were recruited over a period of 12 months. Fifty patients (26 males; 24 females; mean age 64.44, SD 10.52 years) from 285 AIS patients treated at the stroke unit Department of Neurology and Epileptology met the inclusion criteria, entered the study, and used the device properly—i.e., they were able to record at least one 30-s ECG (Figure 2, flow diagram).

Demographic and clinical features of study group and patients who were not able to use device for unclear reasons are summarized in Table 1. Patients had a history of the following symptoms: 22/50 (44%) patients—history of palpitation; 13/50 (26%)—dyspnea; 17/50 (34%)—fatigue; 16/50 (32%)—tightness/pain in the chest; 6/50 (12%)—syncope; 16/50 (32%)—presyncope; and 25/50 (50%)—dizziness.

### 3.2. Feasibility

The recruitment rate among AIS patients was 26.3% (75/285), which was higher than the target set at ≥20%. The withdrawal rate before the start of the intervention was 26% (20/75), which was higher than the established ≤20%. The withdrawal rate after the start of the intervention was 6% (5/75), which met the established criteria of ≤10%. Different technical issues arose during the study. The most important was the problem of connection between KM pad and the portable tablet computer. The KM pad should be localized close to the microphone of the portable tablet computer to start ECG registration; however, it was difficult for most AIS patients to comprehend this. For some patients, it was difficult to touch the metal electrodes correctly to create the bipolar lead and start ECG registration.

### 3.3. The KM ECG Monitoring

The KM device was used by each of the 50 patients 17.9, SD 7.98 times. There was no sex difference in the number of device use (male: 17.11, SD 7.58 times; female: 18.75, SD 8.47 times; *p* = 0.47 at t-student test). There was no correlation between patient’s age and the number of the device use (*p* = 0.94 at Pearson’s test).

KM device detected at least one 30-s ECG, which was interpreted by algorithm as AF in eight patients (8/50, 16% of study group; 8/285, 2.8% AIS patients; 8/193, 4.1% of non-AF AIS patients) and in 20 of 895 total ECG records (20/895, 2.2%). Fifty-one (51/895, 5.7%) ECG records were interpreted as unclassified. The analysis was impossible in nine (9/895, 1%) of the ECG records, and the time of registration was too short in 10 (10/895, 1.1%) of the ECG records.

There was no correlation between each patient’s total number of ECG records and AF registration (T = −0.135, *p* = 0.25 at Kendall’s tau b test). No correlation between ECG records interpreted by the AliveCor algorithm as AF and history of palpitation (χ^2−^ 0.16, *p* = 0.69 at chi-square test), dyspnea (χ^2−^ 0.90, *p* = 0.34 at chi-square test), fatigue (χ^2−^ 1.96, *p* = 0.16 at chi-square test), chest tightness/pain (χ^2−^ 1.664, *p* = 0.20 at chi-square test), presyncope (χ^2−^ 0.132, *p* = 0.72 at chi-square test), syncope (χ^2−^ 0.002, *p* = 0.96 at chi-square test), or dizziness (χ^2−^ 0.00, *p* = 1.00 at chi-square test) was noticed.

### 3.4. KM Automated Algorithm Versus Cardiologist Adjudication

**Figure 3:**
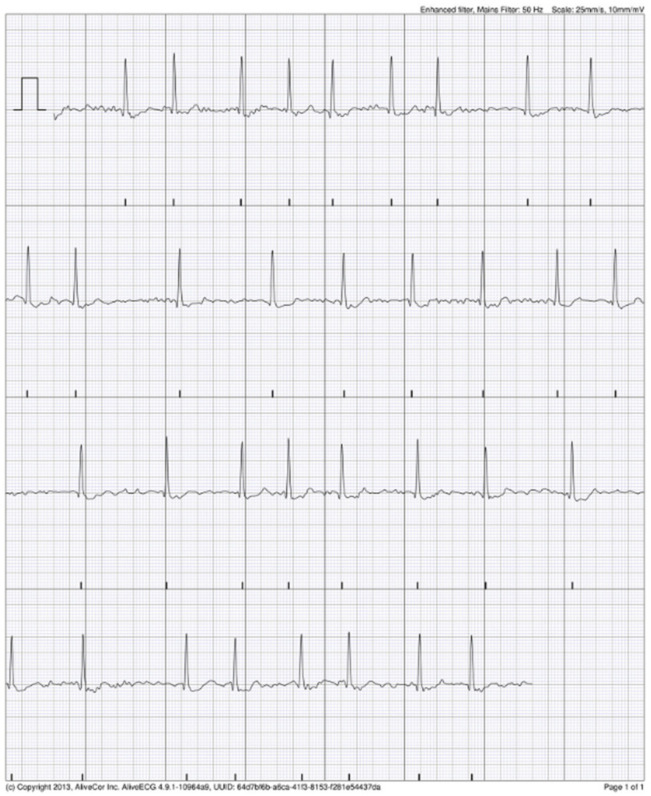
Possible atrial fibrillation (AF) recorded by AliveCor device, as confirmed by cardiologist.

**Figure 4:**
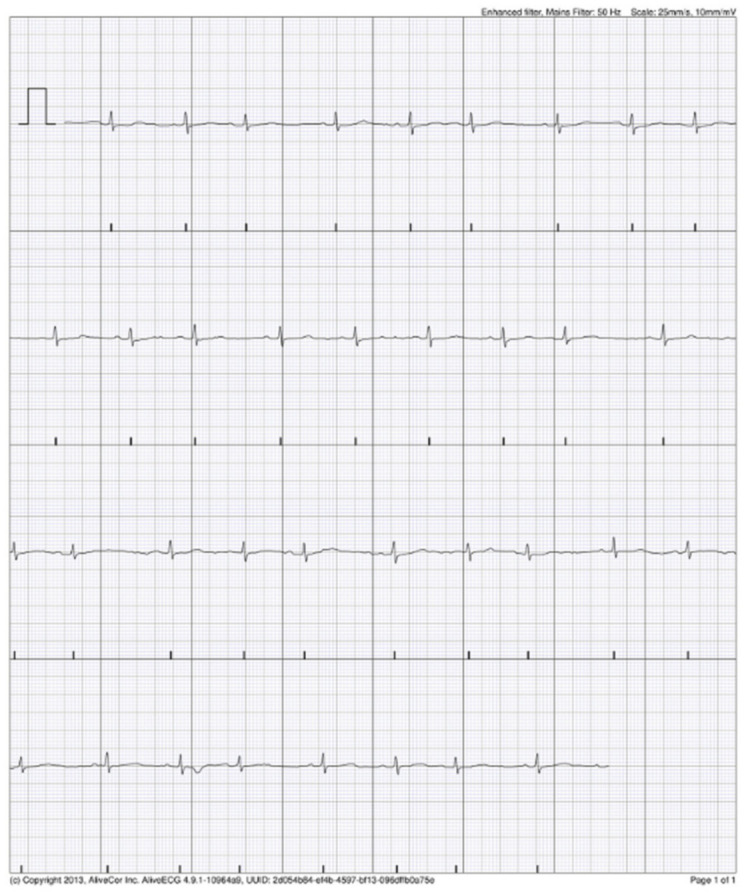
Possible AF recorded by AliveCor device interpreted by cardiologist as supraventricular extrasystoles.

**Figure 5:**
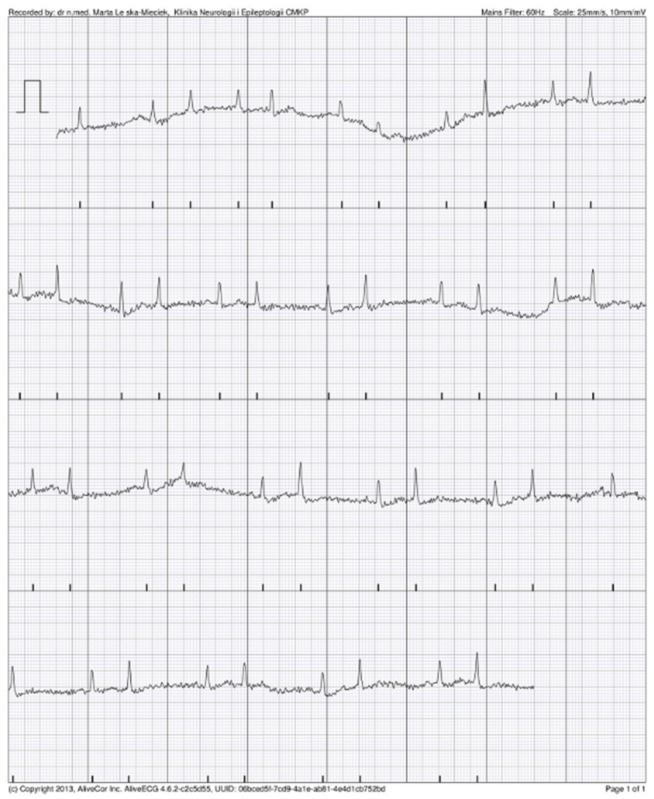
Possible AF recorded by AliveCor device—diagnosis was not confirmed by cardiologist.

**Figure 6:**
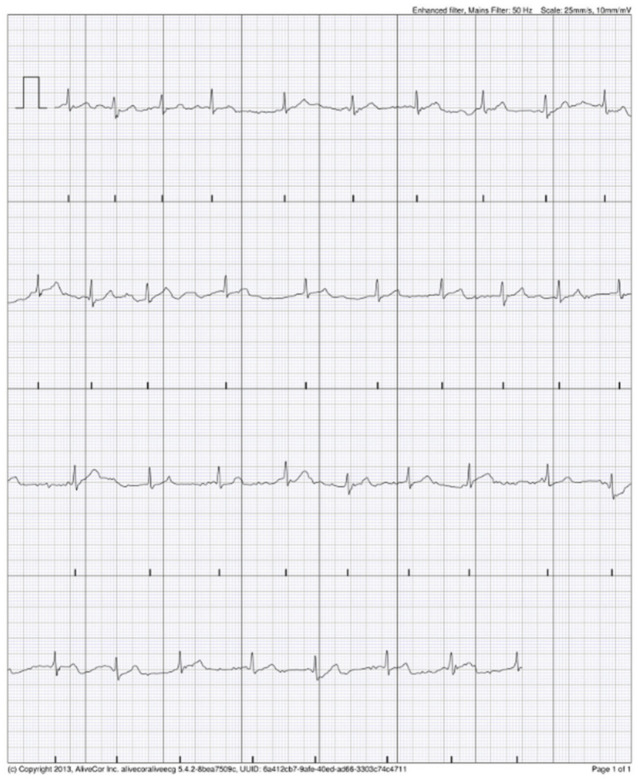
Possible AF recorded by AliveCor device—interpreted by cardiologist as undiagnostic with AF possibility.

Upon review of the single-lead ECG, it was of adequate quality for rhythm decision in 95% (19/20) of ECG records documented by AliveCor algorithm as AF. The cardiologist confirmed the AF in one patient (1/50, 2% of study group; 1/285; 0.3% AIS patients; 1/193, 0.5% non-AF AIS patients) in five KM ECG records (Figure 3). The rest of ECG records documented by AliveCor algorithm as AF were interpreted by cardiologist as supraventricular extrasystoles in three patients in a total of seven KM ECG records (Figure 4). In three patients (seven KM ECG records), the diagnosis of AF was not confirmed by a cardiologist (Figure 5). One KM ECG record interpreted by algorithm as AF was evaluated by a cardiologist as undiagnosed, although AF was a possibility (Figure 6). The data regarding cardiologist’s analysis and 24 h Holter ECG are summarized in Table 2. Sensitivity and specificity of KM for AF were 100% (95% CI 47.8–100%) and 98.3% (95% CI 97.2–99.0%), respectively.

### 3.5. Twenty-Four-Hour Holter ECG

More SVEBs recorded by 24 h Holter ECG in patients with ECG records interpreted by AliveCor algorithm as AF were observed (AF: mean—2183 ± 3287.89, non-AF: mean—256.4, SD 709.64; *p* = 0.01 at Mann–Whitney test). There was no difference in the number of VEBs (AF: mean −200.5, SD 16.55; non-AF: mean—109.17, SD 302.46; *p* = 0.72 at Mann–Whitney test) and SVT (AF: mean −13.38, SD 34.23; non-AF: mean—1.05, SD 2.01; *p* = 0.20 at Mann–Whitney test) recorded by 24 h Holter ECG between patients, whose ECGs were interpreted by AliveCor algorithm as AF and others.

## 4. Discussion

KM has 98.5% sensitivity and 91.4% specificity for AF detection in patients aged 65 years and more [32]. Most of the patients were comfortable with using the device, finding it easy to use, nonrestrictive, and that it did not cause anxiety [33,34]. We found no literature regarding AF monitoring using portable devices without medical staff involvement for AIS patients treated in a stroke unit. According to the present study’s results, selected AIS patients can use the device properly. Feasibility was demonstrated with the achievement of all operationalization criteria, except for the withdrawal rate before starting the intervention. The recruitment rate among AIS patients was 26.3% (75/285), which was higher than the target set at ≥20%. Patients were not able to use the device due to aphasia (32/193, 16.6%), severe neurological condition (17/193, 8.8%), unstable general condition (8/193, 4.1%), visual disturbances (6/193, 3.1%), dementia (5/193, 2.6%), and tremors (2/193, 1%) (Figure 1). There was a group of patients (40/193, 20.7%) who were not able to use the device properly, but the reasons why were difficult to ascertain. Their neurological condition seemed to not be debilitating but they still did not manage to obtain a proper ECG record using the KM device. Patients in that group, as it could be expected, were elderly and in a worse neurological condition than that of the study group (Table 1). The average age of patients in the study group was 64.44 years. AF incidence continues to be much higher in the elderly population, and in adults older than 65 years it is estimated to be 8.6% [35].

The withdrawal rate before the start of the intervention was 26% (20/75), which was higher than the established ≤20%. Five patients were excluded from the study group as the device was not available. The reason was malfunction of the device or an insufficient number of KM pads, which were available for patients (two devices). Fifteen patients finally declined to participate, although they gave preconsent first. All those patients were afraid of accidentally damaging the devices (KM pad and the portable tablet computer), although there were no repercussions to fear.

The withdrawal rate after the start of the intervention was 6% (5/75), which met the established criteria of ≤10%. All five participants had standard training on how to use the KM card and how to record ECG on tablet. They were provided a tablet and KM pad for three days but no ECG record was registered, even though these patients claimed that they used the device regularly and properly. The reason was not a technical one and remains unexplained. It was most likely caused by the patients’ noncompliance.

The technical problem of connection between KM pad and the portable tablet computer was solved using additional headphones attached to portable tablet computer. Patients’ difficulties with touching the KM metal electrodes correctly to create the bipolar lead were the result of neurological deficits. This was solved by fixing the KM pad to the portable tablet computer. Such technical issues can be solved and should not be a reason to preclude the use of KM in selected AIS patients.

The varying population involvement, settings, and clinical scenarios make it difficult to compare results of the published studies on KM device. Further, the automated arrhythmia detection algorithm provided inconclusive results of between 0.8%–27.6% of KM ECGs [36].

In the presented study, across 72 h of screening, patients used the device 17.9 ± 7.98 times. AliveCor algorithm interpreted ECG as possible AF in 16% (8/50) of AIS study patients, 2.8% (8/285) of all AIS, and 4.1% (8/193) of non-AF AIS patients. Halcox et al. reported AF diagnosis in 3.8% participants in high-risk population (CHADS-VASc score ≥ 2) screening using KM device twice weekly over 12 months (plus additional use if symptomatic). Stroke in patients who are found to have clinical AF is one of the most powerful predictors of a recurrent stroke, and this was incorporated into the CHA2DS2-VASc (congestive heart failure, hypertension, age category, diabetes, stroke/TIA/systemic embolism history, sex, vascular disease history) score with two points [33,37]. Proportion of the newly identified AF after stroke are related not only to duration of registration, but also to the timing. The yield of AF detection is highest in the first weeks to months after the cerebral ischemic event, and more intensive monitoring detects more AF [9,38]. When cryptogenic AIS patients are not screened for AF using standard methods, the chance to register AF could be increased by using mobile devices, even at the time of hospitalization.

Although the recruitment rate in the AIS patients (26.3%) was relatively low compared to that of the other screening studies (76.1%), the AF detection rate (4.1% of non-AF patients) was similar to that of the other populations screened with mobile ECG (0.12–8%) [14,39,40]. The detection rate depends on the characteristics of the population being studied and was higher for the hospitalized patients with an increased risk of AF. The results of the presented study were also compared to the results of prolonged, continuous ECG using electrode belt in patients with a recent embolic stroke of unknown source [41]. Using this method in the pilot study, AF was diagnosed in 6.7% of patients. In the presented study, AIS patients able to use mobile ECG were younger and in better neurological condition than that of those excluded. AF diagnosis and proper treatment could help to prevent the next cerebrovascular event and avoid neurological deterioration in relatively independent patients. Diagnosis of patients who have a history of symptoms suggesting AF remains difficult. The only way to establish the underlying heart rhythm is to capture an ECG while the patient has symptoms. Many patients go for years without diagnosis due to the difficulty in capturing the underlying heart rhythm. The advantage of the KM is that patients could capture the heart rhythm at the time of AF symptoms [42,43]. In our study, we found no correlation between KM AF detection and the history of AF clinical symptoms in AIS patients. AF screening using KM could be useful for both symptomatic and asymptomatic AIS patients.

The AliveCor app rhythm analysis algorithm automatically reported any ECG recorded as normal, possible AF, or unclassified. The ECG should be reviewed by consultants, and the therapeutic decision should be made based on their opinion. In the present study, the cardiologist confirmed AF in one patient for every five ECG records. Ninety-five percent of these single-lead ECGs were of adequate quality for a rhythm decision. Pitman et al. reported 89.61% single-lead ECGs to be an adequate quality for a rhythm decision [44]. Although the correlations between extrasystoles and bigeminy were reported by the cardiologist and ECG interpreted by AliveCor algorithm as AF, the observed sensitivity and specificity of KM for AF in the selected high-risk group of AIS patients was 100% and 98.3%, respectively. This confirms data from other studies that reported its sensitivity (66.7–98.5%) and specificity (91.4–97%) [32,45,46,47,48,49]. The clinical outcome of the present study was AF diagnosis leading to therapeutic decisions and to a change in antithrombotic strategy from antiplatelets to anticoagulation in one AIS patient (1/50, 2% of study group; 1/285, 0.3% AIS patients; 1/193, 0.5% non-AF AIS patients). Considering the short duration of the observation time for each patient (three days), it seems to be a simple method to enhance diagnostic efficacy in selected AIS patients. Early diagnosis of AF provides the opportunity for early initiation of anticoagulation treatment to reduce stroke risk and other complications. Data from the literature also point to the role of statins in the treatment of AF-related stroke patients. Poststroke statin therapy reduced the risk of all-cause mortality [50]. In-hospital mortality is significantly higher in the AF-related stroke patients not taking statins before hospitalization than in those who did [51]. In the presented study, the AF screening was made within first nine days after admission to the stroke unit because of the AIS diagnosis (Table 1).

There are no data in the literature regarding AIS patients using smartphone electrographic screening tools while in the hospital. In Tu et al., the protocol for KM ECG monitoring is administered by nursing staff at the same frequency as the vital observations of pulse and blood pressure until discharge [52]. In our study, there was no limitation of KM usage, and selected patients used the device independently (without medical staff involvement). There was no correlation between each patient’s total ECG records and AF registration, as we expected.

Regarding limitations of this study, the feasibility of implementing the KM device in AIS patients was relatively low compared to that of other studies. Patients screened in other studies were not neurologically related ones, nor were they stroke patients. Ischemic stroke is the third leading cause of disability globally [53,54]. Even patients with strokes that are considered mild in clinical assessment showed impaired concentration and memory and could have depression [55,56,57]. The prevalence of poststroke cognitive impairment ranges from 20–80%, which varies between countries, races, and diagnostic criteria [58]. AF increases risk of cognitive decline [59,60]. Those factors, which could affect ability to use KM device, were not investigated in the present study. It is difficult to assess cognitive function in early days of a stroke.

The duration of monitoring was short, as were the repeat intermittent screenings (three days). It was dictated by average length of AIS patient’s hospital stay and another diagnostic method’s schedule (for example, Holter ECG).

The selection bias was that the exclusion criteria for the study allowed only people who could use the KM device. There were 38.9% (75/193) of such participants in the high-risk group of non-AF AIS patients.

AIS patients included in the study were relatively young—64.4 years old, on average. Although some risk factors are the same for the embolic and thrombotic strokes, AF is leading cause of stroke among older patients (>75 years) [61,62]. In the present study, 40 elder patients (75 years old) were excluded from the study because they were unable to use KM device for unclear reasons. The age difference between those two groups was statistically significant (*p* < 0.001) (Table 1).

## 5. Conclusions

The results of this study suggest that it is feasible to use KardiaMobile (KM) device to detect atrial fibrillation (AF) in the selected acute ischemic stroke (AIS) patients (26.3% of AIS). These selected patients were younger (mean age 65 years, *p* < 0.001) and in better neurological condition (Scandinavian Stroke Scale, *p* < 0.001) than that of the remaining AIS patients, who were not able to use the device for unclear reasons (mean age: 75 years). KM detected AF in selected AIS patients with high specificity and sensitivity (100% and 98.3%, respectively).

## Figures and Tables

**Table 1:** Summary of baseline characteristics of study cohort and group of patients not able to use device for unclear reasons.

	Study Group (*n* = 50)	Patients Not Able to Use Device for Unclear Reason (*n* = 40)	*p* Value
Age, mean (year)	64.44 (SD 10.52)	75.15 (SD 11.38)	<0.001
Gender, female (%)	24/50 (48%)	26/40 (65%)	0.11 ^1^
SSS0, mean ^2^	47.10 (SD 11.6)	36.33 (SD 11.88)	<0.001
SSS7, mean ^3^	55.76 (SD 3.74)	45.11 (SD 11.97)	<0.001
SSSE, mean ^4^	55.98 (SD 3.39)	46.83 (SD 11.65)	<0.001
Duration of hospitalization, mean (days)	9.0 (SD 1.23)	11.28 (SD 8.94)	0.22

^1^ Pearson’s chi-square test; ^2^ Scandinavian Stroke Scale (1st day after admission to the stroke unit); ^3^ Scandinavian Stroke Scale (7th day after admission to the stroke unit); ^4^ Scandinavian Stroke Scale (on the last day of the hospital stay).

**Table 2:** Summary of cardiologist’s inspection of KM ECG records and data of standard 24-h Holter ECG registration.

Results of the Cardiologist Inspection of the KM ECG Records		
	Number of Patients (*n* = 50)	Number of KM ECG Records (*n* = 895)
Extrasystoles	4/50 (8%)	13/895 (1.4%)
Bigeminy	1/50 (2%)	3/895 (0.3%)
Bundle branch block	5/50 (10%)	
**Data of the standard 24 h Holter ECG**		
	**Number of patients (*n* = 50)**	**Number of detected arrhythmias, mean**
SVEB ^1^	28/50 (56%)	564 (SD 1573.13)
VEB ^2^	22/50 (44%)	124.08 (SD 341.04)
SVT ^3^	19/50 (38%)	3.02 (SD 13.84)

^1^ SVEB: supraventricular ectopic beat; ^2^ VEB: ventricular ectopic beat; ^3^ SVT: supraventricular tachycardia.

## Data Availability

The data presented in this study are available on request from the corresponding author.
